# Flat Magnetic Stimulation for Stress Urinary Incontinence: A Prospective Comparison Study

**DOI:** 10.3390/bioengineering10030295

**Published:** 2023-02-26

**Authors:** Matteo Frigerio, Marta Barba, Alice Cola, Giuseppe Marino, Silvia Volontè, Tomaso Melocchi, Desirèe De Vicari, Serena Maruccia

**Affiliations:** 1 Department of Gynecology, ASST Monza, San Gerardo Hospital, 20900 Monza, Italy; 2 Gynecology and Obstetrics Department, University of Milano-Bicocca, 20126 Milano, Italy; 3 Department of Urology, ASST Santi Paolo e Carlo, San Paolo Hospital, 20142 Milano, Italy

**Keywords:** magnetic stimulation, stress urinary incontinence, pelvic floor, quality of life, ultrasound

## Abstract

Background: Flat Magnetic Stimulation (FMS) is characterized by a stimulation generated by electromagnetic fields with a homogenous profile. One possible application is the treatment of stress urinary incontinence (SUI). We aimed to compare the objective, subjective, quality of life, and instrumental outcomes in women with SUI not eligible for surgery undergoing either FMS or pelvic floor muscle training (PFMT). Methods: This was a prospective interventional study. After proper counseling, patients with isolated SUI were divided according to their treatment of choice into FMS and PFMT groups. At baseline and after treatment, patients completed the International Consultation on Incontinence Questionnaire-Short Form, the Female Sexual Function Index, and the Incontinence Impact Questionnaire, and volumetric measurement of the urethral rhabdosphincter (RS) was performed. The Patient Global Impression of Improvement questionnaire and stress test defined subjective and objective cure rates, respectively. Results: We observed improvements in urinary-related quality of life scores and an increase in RS volume after FMS compared to baseline. All these outcomes were significantly better compared to women who underwent PFMT. Conclusion: Our study demonstrated that FMS is a safe and effective conservative option for SUI management in terms of objective and subjective cure rates.

## 1. Introduction

Pelvic floor disorders (PFDs) represent a series of conditions—including prolapse, bowel, sexual, and bladder dysfunction—related to pelvic floor weakening and/or tears, usually related to obstetric trauma [1,2]. Additionally, changes in connective tissue composition and metalloproteinases can be observed in patients with pelvic floor disorders [3]. Pelvic floor disorders share the same factors and may frequently coexist or recur [4,5]. Moreover, the treatment of one of these disorders can improve, worsen, or even predispose to another. For example, prolapse repair has been shown to improve overactive bladder symptoms, but worsening has been demonstrated when a concomitant sling procedure is performed at the time of surgery [6]. Among pelvic floor disorders, stress urinary incontinence is considered one of the most bothering conditions. Stress urinary incontinence (SUI) is defined as involuntary leakage of urine during effort, coughing, or sneezing which generally occurs when the intra-abdominal pressure exceeds the urethral closure pressure [7]. This can occur as a consequence of the damage to the connective support of the urethra and bladder due to vaginal delivery, leading to insufficient urethral support [8]. In addition, the changes in collagen composition of the endopelvic fascia and the impairment of the urethral sphincter, related to the menopausal decrease in estrogen, can lead to a reduction in the urethral closure pressure [9]. Moreover, SUI can occur or persist as a consequence of pelvic floor surgery [10,11]. Globally, SUI is estimated to affect up to 50% of women in developed countries and has a lifetime risk of requiring surgery of about 4% [12,13]. Moreover, this condition negatively affects social, occupational, domestic, and psychophysical well-being [14]. Diagnostic confirmation may involve urodynamic evaluation due to the well-established poor correlation between clinics and instrumental findings in bladder dysfunctions [15,16]. However, the role of urodynamics is currently under debate due to different definitions and inconstant performance [17,18]. Management can vary from conservative to surgical treatment according to the severity of symptoms, their impact on quality of life, and the patient’s medical history and comorbidities. Surgical treatment is indicated when conservative management fails. Many types of surgery have been proposed over the years, including bladder neck suspension, anterior vaginal wall repair, autologous sling, stem cell injection, urethral bulking agents, and suburethral tapes [19,20,21,22,23]. However, each surgical approach has its own drawbacks, including visceral injuries (such as bladder perforation) and chronic neurological pain [24,25]. 

Consequently, conservative treatments should represent the first therapeutic approach. These include lifestyle modification, pelvic floor muscle training (PFMT), biofeedback/electrical stimulation, and vaginal laser [26]. Additionally, magnetic stimulation (MS) is considered a conservative treatment option for SUI. MS is a non-invasive therapeutic device that interacts with the neuromuscular tissue through a specific electromagnetic field, inducing intense contractions (involuntary and otherwise unachievable regular gym training or superficial electrical stimulation) that stimulate pelvic floor muscles deep down and restoring neuromuscular control. Various clinical trials have evaluated the efficacy of MS in ameliorating female SUI with positive outcomes [27]. However, due to heterogeneous results and weak evidence of the short-term and long-term effects, current European Urology Association recommendations advise against treating urinary with magnetic stimulation [28]. 

Recently, technological progress has provided advancements in magnetic stimulation equipment. In particular, Flat Magnetic Stimulation (FMS) is characterized by a stimulation generated by electromagnetic fields with a homogenous profile, which can be optimized for the treatment of the pelvic area. The homogeneity of magnetic field distribution does not generate areas of variable stimulation intensity, so the muscle works at the same intensity in all the fields. An advantage of this technology—due to the greater homogeneity of magnetic field distribution in a broader area—is that it allows greater recruitment of muscle fibers without creating areas of variable stimulation intensity. This is thought to be associated with greater treatment efficacy compared with standard MS. The interaction with the tissue involves muscular contraction, depolarization of neuronal cells, and enhancement of the blood circulatory system. Electric currents depolarize the nerve fibers, thus causing concentric contractions that lift all the pelvic muscles. The main effectiveness comes from electromagnetic energy, deep penetration, and stimulation of the entire pelvic floor area. This directly modifies muscle structure, inducing more efficient growth of myofibrils (muscle fiber hypertrophy) and the creation of new protein strands and muscle fibers (fiber hyperplasia muscle). While this new technology may enhance the outcomes of MS, up-to-date data on its efficacy on SUI are scarce. We hypothesize that new FMS may provide results comparable to other conservative treatments in the management of SUI.

As a consequence, with this study, we primarily aimed to compare the objective, subjective, quality of life, and instrumental outcomes in women suffering from stress urinary incontinence not eligible for surgery undergoing either FMS or PFMT. As a secondary outcome, we wanted to evaluate the effects on sexual function.

## 2. Materials and Methods

This was a prospective interventional study. Recruitment occurred from gynecologic outpatients in San Gerardo Hospital in Monza from August 2022 to September 2022. In the period of interest, all patients underwent a clinical interview to investigate the presence of lower urinary tract symptoms, including stress urinary incontinence (SUI), overactive bladder (OAB), urge urinary incontinence (UUI), voiding symptoms (VS), bulging symptoms or fecal incontinence. All definitions conformed to International UroGynacology Association/International Continence Society terminology [7]. In addition, a urogenital examination was carried out and descensus staged according to the Pelvic Organ Prolapse Quantification (POP-Q) system. 

To be considered eligible for the study, patients should have isolated SUI without surgical indication, confirmed with a standard 300 mL stress test [29]. Exclusion criteria were women <18 years old, with insufficient Italian language proficiency, in a state of pregnancy, with an implanted pacemaker, defibrillator, neurostimulation, or ferromagnetic prostheses, weighing >160 kg, with recent deep venous thrombosis, fever, acute inflammatory diseases or recent fractures in the area of treatment, neoplasia, arrhythmia, and congestive heart failure. At the baseline, all patients completed the International Consultation on Incontinence Questionnaire-Short Form questionnaire (ICIQ-SF), the Female Sexual Function Index (FSFI-19) questionnaire, and the Incontinence Impact Questionnaire (IIQ-7) [30,31,32]. The ICIQ-SF is a robust tool to measure the frequency, severity, and impact of incontinence on quality of life among all patient types [30]. The questionnaire comprises four major questions, with the first three adding up to yield the total score: the frequency of leakage, the perceived quantity of leakage, and the degree of interference with life [30]. The fourth item, which is not considered in the scoring system, is a self-diagnostic item to identify the specific type of incontinence [30]. This tool has been demonstrated to have high levels of validity, reliability, and sensitivity, estimated according to standard psychometric methods [30]. The FSFI-19 is a 5-point Likert scale self-reported questionnaire with 19 items covering six domains of sexual function (sexual desire, lubrication, arousal, orgasm, pain, and satisfaction). FSFI-19 is one of the most popular, powerful, and useful diagnostic tools for investigating female sexual dysfunction and monitoring the efficacy of the treatment [31]. The scale has been tested to evaluate the impact of diverse clinical conditions and treatments on sexual dysfunction and has consistently demonstrated excellent psychometric properties [31]. An FSFI total score of 26.5 has been found to be the optimal cut-off for differentiating women with and without sexual dysfunction [31]. The IIQ-7 was developed to assess the impact on life of urinary incontinence among women [32]. This consists of seven items referring to the individual’s perceived impact of urinary incontinence on daily activities, relationships, and feelings [32]. Each item has a four-point response scale where individuals rate the extent to which urine leakage affects their daily functioning in four domains: physical activity (items 1 and 2), travel (items 3 and 4), social activities (item 5), and emotional health (items 6 and 7) [32]. Over time, this tool demonstrated an excellent degree of acceptability, reliability, and validity across different countries and cultures [32].

**Figure 1:**
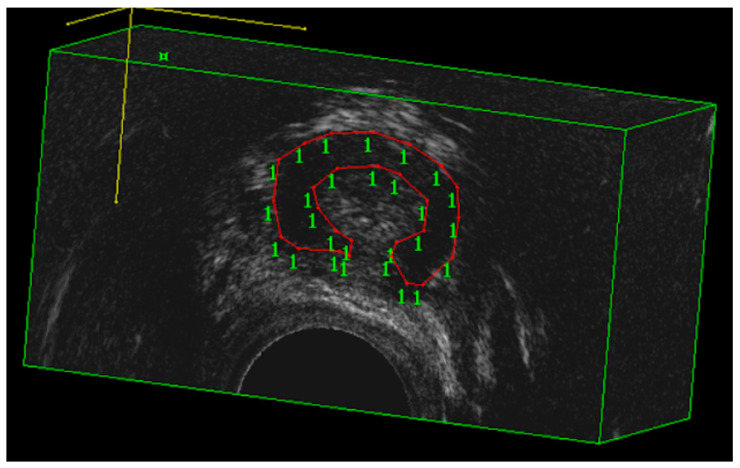
Volumetric assessment of the urethral rhabdosphincter (RS) using a BK Flex Focus 400 sonographic machine equipped with a 9052 transducer by vaginal approach.

In addition to the quality-of-life tools, a volumetric assessment of the urethral rhabdosphincter (RS) was performed using a BK Flex Focus 400 sonographic machine equipped with a 9052 transducer by vaginal approach (BK Medical, Melegnano, Italy). This is a mechanical, single-element, multifrequency transducer with a built-in 3D acquisition system providing a 360° field of view over a longitudinal distance of 60 mm. Obtained volumes were assessed offline using the BK 3D Viewer 7.1 software in cubic mode. In this mode, the operator is able to perform volume measurements by delineating the margin of the RS on successive planes to achieve volume values (Figure 1).

After proper counseling, patients were divided according to their treatment of choice into a Magnetic Stimulation (MS) group and Pelvic Floor Muscles Training (PFMT) group. Magnetic stimulation treatment was carried out twice a week for one month involving 8 sessions of 25 min each with Dr. Arnold (DEKA, Calenzano, Italy). The following FMS protocol was applied. Sessions 1 to 4 followed the Hypotonus/Weakness 1 protocol. Sessions 5 to 8 followed the Hypotonus/Weakness 2 protocol. 

Hypotonus/Weakness 1 protocol consists of a Warm-up and muscle activation phase, a Muscle work aimed at recovering tropism and muscle tone phase (20-30Hz) in a Trapezoidal shape for a total time of 25 minutes. Hypotonus/Weakness 2 protocol consists of a Warm-up and muscle activation phase, a Muscle work aimed at increasing tropism (volume) and muscle strength phase (40-50Hz) in a Trapezoidal shape for a total time of 25 minutes. 

Pelvic floor muscle training was performed for one month autonomously at home by patients following the Italian version of the International Urogynecological Association (IUGA) dedicated leaflets [33].

At the end of the treatment, the objective cure rate was assessed with a 300 mL stress test. The ICIQ-SF, FSFI-19, and IIQ-7 questionnaires were collected again, and quality of life outcomes were determined as the difference between preoperative and postoperative questionnaire scores. The subjective cure rate was determined by the Patient Global Impression of Improvement (PGI-I) questionnaire [34], and subjective success was defined as an improvement in the PGI-I score (≤3). The sonographic evaluation was repeated, and the instrumental outcome was determined as the difference between preoperative and postoperative RS volumes.

The study obtained local Ethics Committee approval (protocol code PF-MAGCHAIR). Statistical analysis was performed using JMP software version 9 (SAS Institute, Cary, NC, USA). Outcomes are reported as mean ± standard deviation for continuous variables and as number (percentage) for noncontinuous variables. Differences were tested using paired T-test for continuous parametric data, Wilcoxon test for continuous non-parametric data, and Fisher’s test for non-continuous data. A *p*-value < 0.05 was considered statistically significant. 

## 3. Results

A total of 50 patients were enrolled. Overall, 25 patients underwent magnetic stimulation, whereas the remaining 25 women underwent pelvic floor muscle training at home following the IUGA leaflet. The patients’ characteristics are shown in Table 1. No differences were found in terms of age, parity, or BMI. Baseline (T0) urogenital symptoms severity according to IIQ-7, ICIQ-SF, and FSFI-19 scores were similar. Moreover, baseline 3D ultrasound evaluation demonstrated similar urethral rhabdosphincter volumes between the MS and PFMT groups (*p* = 0.848). During the treatment, no adverse effects were reported. Post-treatment (T1) objective, quality of life, and ultrasound outcomes are reported in Table 2. Rates of urinary leakage during stress tests recorded a 40% decrease for FMS (*p* < 0.001), whereas no improvement was observed for PFMT compared to baseline. An improvement in IIQ-7 (20.7 vs. 33.7; *p* < 0.001) and ICIQ-SF (8.3 vs. 11.2; *p* = 0.003) scores was observed after FMS compared to baseline, whereas non-significant changes were observed in PFMT patients. Sexual function, according to FSFI-19 was not affected by either FMS or PFMT. The instrumental evaluation demonstrated a significant increase in urethral RS (2.5 cm^3^ VS 2.9 cm^3^; *p* < 0.001) after FMS, but this parameter was not affected by PFMT (2.5 cm^3^ VS 2.6 cm^3^; *p* = 0.248). The comparison between post-treatment outcomes of FMS and PFMT (Table 3) showed a significative superiority of the former in terms of objective (40% vs. 0%; *p* < 0.001) and subjective cure rate (72% vs. 20%; *p* < 0.001), IIQ-7 (*p* = 0.002) and ICIQ-SF (*p* = 0.024) scores, and RS volume (2.6 cm^3^ vs. 2.9 cm^3^; *p* < 0.001).

## 4. Discussion

Our study demonstrated that FMS is a safe and effective conservative option for SUI management, in terms of objective and subjective cure rate. Moreover, we observed improvements in urinary-related quality-of-life scores and an increase in RS volume after FMS compared to baseline. Lastly, all these outcomes were significantly better compared to women who underwent PFMT.

Recently, the necessity to offer high efficacy–low morbidity treatment options for the management of SUI has become more and more important. From a surgical point of view, this contributed to the development and widespread adoption of new minimally invasive techniques, such as urethral bulking agents and single-incision slings. The first procedure consists of injections of an agent (such as polyacrylamide hydrogel) into the submucosal tissues of the urethra to increase the coaptation of the urethral walls, leading to increased urethral resistance and improved continence. The principal advantages of this surgical strategy are the reduced rate of adverse events and the chance of proposing these procedures to patients with severe comorbidities. Single-incision slings (SISs) are characterized by shorter tape length and consequently, a limited intracorporeal dissection and lack of full passage of the introducers through the obturator foramen, adductor tendons, and skin. This results in a lower risk of complications, including visceral injury, major bleeding, infection, and neurological pain, shorter recovery time, and a negligible learning curve [35,36]. While excellent short-term efficacy rates unaffected by age, BMI, obstetrical history, and proper bilateral anchoring on obturator membranes have been demonstrated, long-term data is scarce [37,38]. However, despite surgical innovations, surgical strategies always involve a certain—even if minimal—risk of complications.

Consequently, most guidelines recommend conservative management as the first-line treatment for SUI. Different options include PFMT, biofeedback, functional electrical stimulation, and MS but the evidence, including comparative studies, is scarce. Among all conservative treatment options, MS offers some advantages. Patients with pelvic floor disorders may have difficulty performing isolated voluntary pelvic floor muscle contractions. Consequently, the effectiveness of PFMT may be impaired because the patient is not performing it correctly and consistently over time [39]. Moreover, PFMT has the disadvantage of slow progression, patients’ low compliance, and low adherence rates [40]. Both biofeedback and functional electrical stimulation involve the use of an endocavitary probe, which can greatly reduce compliance. Moreover, with electrical stimulation, half of the patients report various degrees of side effects with treatment, the majority of which are related to local discomfort, and 12% of the patients discontinued treatment [41]. Lastly, vaginal habitability may be impaired by a series of conditions, such as previous radiation therapy, previous pelvic surgery, or lichen sclerosus. MS has the advantages of being a passive rehabilitation that does not require the use of vaginal probes, patients do not need to undress, and no adverse effects are expected. Moreover, unlike the electrical current, the conduction of magnetic energy is unaffected by tissue impedance. Consequently, it can be considered a safe, non-invasive, and painless alternative option for the treatment of stress urinary incontinence.

Over the years, many studies demonstrated the role of MS in treating urinary incontinence. However, the differences among them in terms of stimulus intensities, frequencies, locations, and durations and the lack of standardization of the protocols caused the EUA to advise against MS for urinary incontinence treatment. However, reports show encouraging results in terms of MS efficacy for SUI treatment. For example, a randomized controlled trial conducted by Weber-Rajek et al. assessed the physical and psychosocial functioning of 128 women with stress urinary incontinence following MS or PFMT. In this study, the authors concluded that pelvic floor muscle training and extracorporeal magnetic innervation proved to be effective treatment methods for stress urinary incontinence in women [42]. Another randomized study conducted on 120 patients with SUI suggested that active MS treatment significantly improved limitations in physical activities and feelings of depression both immediately after and at 1-year post-treatment, compared with the sham group [43]. 

Our study confirmed that FMS is safe and effective in the short term in treating SUI, in terms of objective and subjective cure rates. Moreover, we observed improvements in urinary-related quality of life scores and an increase in RS volume after FMS compared to baseline. Lastly, all these outcomes were significantly better compared to women who underwent PFMT. FMS technology represents the latest innovation in magnetic stimulation technology. FMS triggers intense muscular contractions inducing electric stimulus by targeting the neuromuscular tissue in the pelvic floor area. This is expected to change the muscular structure, inducing hypertrophy and hyperplasia. Initial evidence of this kind of device seems to be very promising for the treatment of SUI, overactive bladder, and mixed urinary incontinence. For example, Lopopolo et al. reported a significant improvement in quality of life and patients awareness of the pelvic floor area in 50 women with mixed urinary incontinence treated with six sessions of FMS even at the end of the treatment and at three months follow-up. In addition, at the baseline evaluation, patients most frequently experienced leakage several times a day, whereas after six sessions, the leakage occurred only about once a week or less [44]. Biondo et al. evaluated the effectiveness and safety of flat magnetic stimulation in eighty-one female patients (35 patients who reported SUI symptoms and 46 patients who reported UUI symptoms) after eight 28 min treatment sessions (twice a week for 4 weeks). Two questionnaires were used to evaluate the urinary improvements: Incontinence Questionnaire Overactive Bladder Module (ICIQ-OAB) for patients with UUI, and Incontinence Impact Questionnaire—Short Form (IIQ-7) for patients with SUI. According to questionnaire results, both improvements in UUI and SUI symptoms were observed; in particular, IIQ-7′s average score significantly decreased (*p* < 0.05) from 15.53 ± 5.62 at baseline to 6.76 ± 3.10 at 3-month follow-up [45]. Literature suggests that successful treatment of SUI can improve overall female sexual function scores. However, we did not observe improvement in sexual function according to FSFI-19 scores. These may be explained by the low prevalence of sexually active patients in our population, as well as by an underpowered sample for this outcome.

Ultrasound evaluation of RS volume as a marker of MS efficacy represents an original contribution of our study. Previous experiences demonstrated the feasibility and reproducibility of this measure [46,47]. Moreover, this volume has been demonstrated to be greater in continent women than women with genuine stress incontinence and may also play a prognostic role in anti-incontinence surgery outcomes [48,49]. Our study demonstrated significant RS hypertrophy as an effect of FMS, resulting in a 15.4% increase in muscular volume. FMS technology was previously reported to have a similar effect on other skeletal muscles. The efficacy of Schwarzy (DEKA MELA) has been evaluated on the abdomen of 15 patients in a study conducted by Leone et al. This paper demonstrated hypertrophy in terms of abdominal muscle tissue thickness 1 month after the last treatment in all treated areas: upper abdomen (11 ± 1 mm vs. 9 ± 2 mm ), lower abdomen (13 ± 2 mm vs. 10 ± 2 mm vs.), lateral abdomen (13 ± 3 mm vs. 11 ± 2 mm vs.), and rectus abdominis diastasis (25 ± 4 mm vs. 22 ± 4 mm), which are consistent with our findings on urethral RS volumes [50].

To the best of our knowledge, this is the first study evaluating the outcomes in patients with isolated SUI treated with FMS. Strengths include the PFMT comparison group, the high adherence rate with no loss at follow-up, and the multimodal evaluation of outcomes. In addition, ultrasound evaluation of urethral rhabdosphincter represents an original evaluation of FMS efficacy, which can be potentially used to evaluate the efficacy of the other conservative option for SUI treatment. Limitations involve the short-term follow-up, the likely underpowered sample for sexual outcomes, and the lack of randomization. A medium-term follow-up study is currently ongoing at our Institution.

## 5. Conclusions

Our study demonstrated that FMS is a safe and effective conservative option for SUI management in terms of objective and subjective cure rate. Moreover, we observed improvements in urinary-related quality of life scores and an increase in RS volume after FMS compared to baseline. Lastly, all these outcomes were significantly better compared to women who underwent PFMT.

## Figures and Tables

**Table 1:** Population characteristics and baseline (T0) findings. FMS = magnetic stimulation. PFMT = pelvic floor muscle training. Continuous data as mean ± standard deviation. Non-continuous data as absolute (relative) frequency.

	FMS	PFMT	*p* Value
Age (years)	60.9 ± 12.7	60.2 ± 12.7	0.851
Parity (n)	1.9 ± 0.7	2.1 ± 0.7	0.327
BMI (kg/m^2^)	25.4 ± 3.0	25.6 ± 2.9	0.964
T0 IIQ-7 score	33.7 ± 22.6	38.1 ± 14.8	0.318
T0 ICIQ-SF score	11.2 ± 3.6	11.0 ± 3.1	0.814
T0 FSFI-19 score	12.5 ± 11.2	10.9 ± 10.6	0.622
T0 Urethral rhabdosphincter volume (cm^3^)	2.5 ± 0.9	2.5 ± 0.6	0.848

**Table 2:** Pre- and post-treatment comparisons. FMS = magnetic stimulation. PFMT = pelvic floor muscle training. Continuous data as mean ± standard deviation. Non-continuous data as absolute (relative) frequency. T0 = baseline; T1 = after treatment.

	FMS			PFMT		
	T0	T1	*p* Value	T0	T1	*p* Value
Negative stress test	0 (0%)	10 (40%)	**<0.001**	0 (0%)	0 (0%)	1.000
IIQ-7 score	33.7 ± 22.6	20.7 ± 18.7	**<0.001**	38.1 ± 14.8	36.3 ± 14.9	0.119
ICIQ-SF score	11.2 ± 3.6	8.3 ± 4.1	**0.003**	11.0 ± 3.1	10.7 ± 3.2	0.129
FSFI-19 score	12.5 ± 11.2	13.2 ± 11.5	0.463	10.9 ± 10.6	10.0 ± 9.0	0.416
URS volume (cm^3^)	2.5 ± 0.9	2.9 ± 1.1	**<0.001**	2.5 ± 0.6	2.6 ± 0.6	0.248

**Table 3:** Post-treatment outcomes. FMS = magnetic stimulation. PFMT = pelvic floor muscle training. Continuous data as mean ± standard deviation. Non-continuous data as absolute (relative) frequency.

	FMS	PFMT	*p* Value
Negative stress test	10 (40%)	0 (0%)	**<0.001**
PGI-I ≤ 3	18 (72%)	5 (20%)	**<0.001**
T1 IIQ-7 score.	20.7 ± 18.7	36.3 ± 14.9	**0.002**
T1 ICIQ-SF score	8.3 ± 4.1	10.7 ± 3.2	**0.024**
T1 FSFI-19 score	13.2 ± 11.5	10.0 ± 9.0	**0.308**
T1 Urethral rhabdosphincter volume (cm^3^)	2.9 ± 1.1	2.6 ± 0.6	**<0.001**

## Data Availability

The data presented in this study are available on request from the corresponding author.
